# Transcription factor EB reprograms branched‐chain amino acid metabolism and promotes pancreatic cancer progression via transcriptional regulation of *BCAT1*

**DOI:** 10.1111/cpr.13694

**Published:** 2024-06-27

**Authors:** Ting Wang, Qiangsheng Hu, Borui Li, Guixiong Fan, Desheng Jing, Junfeng Xu, Yuheng Hu, Qin Dang, Shunrong Ji, Chenjie Zhou, Qifeng Zhuo, Xiaowu Xu, Yi Qin, Xianjun Yu, Zheng Li

**Affiliations:** ^1^ Department of Pancreatic Surgery Fudan University Shanghai Cancer Center Shanghai China; ^2^ Department of Oncology, Shanghai Medical College Fudan University Shanghai China; ^3^ Shanghai Pancreatic Cancer Institute Shanghai China; ^4^ Pancreatic Cancer Institute Fudan University Shanghai China; ^5^ Department of Thoracic Surgery, Shanghai Pulmonary Hospital Tongji University School of Medicine Shanghai China; ^6^ Department of Hepatobiliary and Pancreatic Surgery Tenth People's Hospital of Tongji University Shanghai China

## Abstract

Pancreatic cancer cells have a much higher metabolic demand than that of normal cells. However, the abundant interstitium and lack of blood supply determine the lack of nutrients in the tumour microenvironment. Although pancreatic cancer has been reported to supply extra metabolic demand for proliferation through autophagy and other means, the specific regulatory mechanisms have not yet been elucidated. In this study, we focused on transcription factor EB (TFEB), a key factor in the regulation of autophagy, to explore its effect on the phenotype and role in the unique amino acid utilisation pattern of pancreatic cancer cells (PCCs). The results showed that TFEB, which is generally highly expressed in pancreatic cancer, promoted the proliferation and metastasis of PCCs. *TFEB* knockdown inhibited the proliferation and metastasis of PCCs by blocking the catabolism of branched‐chain amino acids (BCAAs). Concerning the mechanism, we found that TFEB regulates the catabolism of BCAAs by regulating BCAT1, a key enzyme in BCAA metabolism. BCAA deprivation alone did not effectively inhibit PCC proliferation. However, BCAA deprivation combined with eltrombopag, a drug targeting TFEB, can play a two‐pronged role in exogenous supply deprivation and endogenous utilisation blockade to inhibit the proliferation of pancreatic cancer to the greatest extent, providing a new therapeutic direction, such as targeted metabolic reprogramming of pancreatic cancer.

## Graphical abstract

TFEB, a key regulator of autophagy, reprograms the metabolism of BCAAs by transcriptionally regulating the BCAT1 and thus influences pancreatic cancer progression. Although the usage of BCAAs is essential for pancreatic tumour to progress, BCAAs deprivation alone did not effectively inhibit the proliferation of pancreatic cancer cells. However, BCAA deprivation combined with the use of eltrombopag, a drug targeting TFEB, can play a two‐pronged role in exogenous supply deprivation and endogenous utilisation blockade to inhibit the proliferation and development of pancreatic cancer to the greatest extent, providing a new direction for new therapeutic methods, such as targeted metabolic reprogramming of pancreatic cancer.

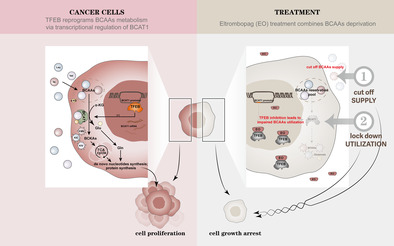

## 1 INTRODUCTION

Pancreatic cancer is a highly malignant cancer with increasing incidence owing to the ageing of society, the growing rates of obesity, diabetes in the population and lifestyle changes including alcohol and tobacco abuse.^1^ Due to its deep location and insufficient early clinical manifestations, it often progresses to an advanced stage, and the opportunity for resection is lost. Other treatments have limited efficacy, poor prognoses and very low five‐year survival rate.^2^ In general, current treatment methods still need to be explored; therefore, the mechanism and clinical translational research on pancreatic cancer are urgently needed.

Owing to the continuous and rapid proliferation of cancer cells, their metabolic requirements are much higher than those of normal cells to meet their growth needs and proliferation of raw materials. One of the hallmarks of cancer cells is the shift in metabolism from cellular respiration to aerobic glycolysis, known as the Warburg effect, which is a classic example of a metabolic shift in which tumour cells can obtain and metabolise nutrients in a way that favours proliferation rather than efficient ATP production.^3^ In addition to this, cancer cells can alter their metabolic pathways through oncogene activation and epigenetic modifications to supply their increased proliferation and metabolic demands.^4^

Metabolic reprogramming is a major feature of pancreatic cancer that ensures high metabolic demands in a nutrient‐deficient tumour microenvironment.^5^ Compared to other cancer cells, pancreatic cancer cells are in a more nutrient‐limited state due to their characteristic high interstitial pressure and connective tissue hyperplasia.^6^, ^7^ Metabolism using nitrogen sources is an important metabolic feature that distinguishes pancreatic cancer cells from normal pancreatic cells and provides the metabolic conditions necessary for the growth and proliferation of pancreatic cancer.^8^ Pancreatic cancer cells accumulate essential amino acids and consume glutamine to support cancer cell growth^9^; however, the mechanism by which this metabolic transition occurs remains to be studied.

Branched‐chain amino acids (BCAAs), including leucine, isoleucine and valine, are the most abundant essential amino acids and play key roles in energy homeostasis, nutrient metabolism, immunity and the regulation of a variety of diseases in humans and animals.^10^ BCAAs are not raw materials for biosynthesis of nitrogen‐containing compounds but also act as signalling molecules to regulate the anabolism of glucose, lipids and proteins.^10^ BCAAs are indispensable in cancer cells with various physiological and metabolic effects; cancer cells increase the uptake and utilisation of BCAAs, providing indispensable raw materials for cell proliferation.^11^ Elevated levels of BCAA‐metabolising enzymes have long been reported in pancreatic cancer and are thought to play a role in promoting tumour proliferation.^12^ Therefore, targeting the metabolic differences between pancreatic cancer and normal cells is expected to be an ideal strategy for cancer treatment.

Transcription factor EB (TFEB) is a member of the MIT/TFE transcription factor family, which has been found to be able to bind to the coordinated lysosomal expression and regulation (CLEAR) sequence as a transcription factor. The CLEAR motif is enriched in the promoters of lysosomal and autophagy genes and drives the expression of the entire network of genes carrying CLEAR, leading to an increase in the number of lysosomes and autophagosomes and promoting autophagosome‐lysosome fusion.^13^ Therefore, TFEB has the role of centrally regulating lysosomal occurrence, expression and autophagy, which is of great regulatory significance for cell autophagy.^14^ Autophagy can maintain cellular metabolic homeostasis and promote cell survival. The survival and progression of pancreatic cancer depend on autophagy; therefore, the role of TFEB, a key regulator of autophagy, in cancer progression and regulation has been widely studied.^15^, ^16^ Recently, in addition to playing an important role in cancer as a key regulator of autophagy, TFEB was found to play a cancer‐promoting role in an autophagy‐independent manner; however, the mechanism remains to be explored.^17^

In this study, we demonstrated that TFEB in pancreatic cancer cells (PCCs) regulates the BCAA metabolism pathway, which is essential for cell proliferation and metastasis, by regulating BCAT1, a key enzyme in BCAA metabolism. Knockdown of *TFEB* leads to considerable inhibition of PCCs proliferation and migration, which is achieved by downregulating *BCAT1* at the transcriptional level, thereby blocking the breakdown and utilisation of BCAAs. By supplementing BCAA catabolism, the tumour inhibitory effect caused by *TFEB* knockdown can be significantly reversed. The combination of targeted TFEB and BCAT1 or targeted TFEB combined with a low‐BCAA diet may have translational therapeutic significance for PCCs.

## 2 MATERIALS AND METHODS

### 2.1 Cell culture

The human pancreatic cancer cell lines PANC‐1, CFPAC‐1, SW1990, Mia Capa‐2, Aspc‐1 and HPAF‐II were obtained from the American Type Culture Collection (ATCC), HPDE6‐C7 (H6C7) and Capan‐1 were obtained from Cell Bank of Chinese Academy of Sciences. PANC‐1, Capan‐1 and H6C7 cells were cultured with high‐glucose Dulbecco's Modified Eagle Medium (DMEM) supplemented with 10% FBS. Mia Capa‐2 cells were cultured with DMEM supplemented with 10% FBS and 2.5% horse serum. CFPAC‐1 cells were cultured with Iscove's Modified Dulbecco's Medium (IMEM), supplemented with 10% FBS. SW1990 cells were cultured with L‐15 with 10% FBS. Aspc‐1 cells were cultured with RPMI‐1640 with 10% FBS. HPAF‐II cells were cultured with MEM with 10% FBS. All cells were cultured at 37°C in a 5% CO_2_ humidified incubator. Customised BCAA‐free DMEM and IMEM (Livning, Lvn1019 and Lvn1001) were used for branched‐chain amino acid deprivation experiments.

### 2.2 Plasmids

The pLKO.1 TRC cloning vector (Addgene plasmid # 10878) was used to knockdown TFEB, targeting two sequences as follows:

sh TFEB‐1: 5′‐CGATGTCCTTGGCTACATCAA‐3′;

sh TFEB‐2: 5′‐GAACAAGTTTGCTGCCCACAT‐3′.

The coding sequence of human TFEB was cloned into the lentiviral vector pCDH‐CMV‐MCS‐EF 1‐puro (SBI, USA) to generate a TFEB expression plasmid. The coding sequence of human TFEB was cloned into the lentiviral vector pCDH‐CMV‐MCS‐EF 1‐puro (SBI, USA) to generate TFEB expression plasmids. Relative shRNA‐cell and overexpression‐cell pools were selected with puromycin after viral transduction.

### 2.3 Chemicals

TFEB inhibitor Eltrombopag (EO) was purchased from Shanghai Titan Scientific and was treated with a concentration of 10 μM for western blotting, RT‐qPCR, CCK8 assay and colony formation assay. BCKAs including KIV, KIC and KMV were purchased from Sigma‐Aldrich and Macklin. BCAAs including leucine, valine, isoleucine were purchased from abmole. For cell cycle assays, CCK8 and colony formation assays, cells were treated with BCKAs at a concentration of 0.4 mM.

### 2.4 Western blotting

Whole protein lysates were extracted, separated by SDS‐Page, and imprinted onto PVDF membranes (BioRad). After 2 h of milk blocking at room temperature, the membranes were incubated overnight with the first antibody (1:1000), including anti‐TFEB (abcam), anti‐BCAT1 (Biotech, proteintech), β‐actin (abclonal), anti‐flag (abclonal), Phosho‐ERK(CST), ERK(CST), Phospho‐AKT(CST), AKT(CST). Use secondary antibodies conjugated to HRP (abclonal) were incubated for 1.5 h at room temperature, and bands were detected with an enhanced chemiluminescence detection kit (EpiZyme). The western blotting experiments were repeated three times, and typical images from a single repeat are shown.

### 2.5 Tissue specimens and immunohistochemical staining

The clinical tissue samples used in this study are the same as those in previous studies at our centre, with prior informed consent from patients and approval from institutional research ethics committees.^18^ Patient tissue microarray was analysed by immunohistochemical (IHC), and protein expression was detected using anti‐TFEB (1:50, Santa Cruz) and anti‐BCAT1 (1:100, Proteintech). The IHC staining results were determined by the staining area grade (grade 0 to grade 4, from 0% to 100%) multiplying the staining intensity grade (0: negative, 1: weak staining, 2: moderate staining and 3: strong staining) to calculate the IHC score (H‐score) of each specimen. The staining area grade was converting from the staining distribution area, and defined as 0% to 5% as 0, 6% to 25% as 1, 26% to 50% as 2, 50% to 75% as 3 and over 75% as 4.^19^ The median H‐score was used as the cut‐off criterion to group the patients, the survival curve was used to compare the survival of patients with different staining intensities of TFEB and BCAT1, and the spearman correlation analysis was performed by H‐score analysis of TFEB and BCAT1 IHC staining to analyse the gene expression correlation of clinical samples.

### 2.6 RNA extraction and quantitative real‐time PCR

Total RNA was extracted using Universal RNA Extraction Kit (accurate biology). Reverse transcription was conducted using the TaKaRa Primescript RT reagent Kit. Quantitative real‐time PCR was conducted by an ABI 7900HT Real‐Time PCR system (Applied Biosystems). Primers used are as follows,

TFEB: 5′‐ACCTGTCCGAGACCTATGGG‐3′ (Forward),

5′‐ACCTGTCCGAGACCTATGGG‐3′ (Reverse),

BCAT1: 5′‐ACCTGTCCGAGACCTATGGG‐3′ (Forward),

5′‐CCAGGCTCTTACATACTTGGGA‐3′ (Reverse),

β‐actin: 5′‐CACCATTGGCAATGAGCGGTTC‐3’ (Forward),

5′‐AGGTCTTTGCGGATGTCCACGT‐3′ (Reverse).

### 2.7 Dual‐luciferase reporter gene assay

PANC‐1 cells overexpressing TFEB were grown in 96‐well plates, and the BCAT1 promoter region spanning −2500 to +200 across the transcription start site was cloned into the pGL3‐Basic vector. After 24 h of incubation, cells are harvested and luciferase activity is measured.

### 2.8 Chromatin immunoprecipitation assay

Chromatin immunoprecipitation (ChIP) assays were performed using the SimpleChIP Kit (Cell Signaling Technology, 9003S) according to the manufacturer's protocol. The ChIP assays were repeated three times, and typical images from a single repeat are shown. Primers for detecting BCAT1 promoter occupancy are listed below,

Primer: F: 5′‐CTGGAGGCTGAAACCCTTGTCATA‐3′,

R: 5′‐TATCTCCCATACCAAGTGGCCTGC‐3′.

### 2.9 Elisa assays

BCAAs and BCKAs were measured using Elisa kits according to the manufacturer's instructions. Human BCAA Elisa kit was purchased from abcam (#ab83374) and human BCKA Elisa kit was purchased from mlbio (#YJ912536).

### 2.10 Colony formation assay and CCK8 assay

Colony formation assay and cell count kit‐8 (CCK8) assay were used to determine the proliferation ability of cells. For colony formation assay, the indicated cells were seeded in triplicate in each well of six‐well plates, and cultivate for 14–21 days in the incubator to allow colony formation. Colonies were stained with GIEMSA and quantified.

### 2.11 Xenograft animal model

All procedures were authorised by the Medical Ethics Committee of Fudan University Cancer Center. Female mice (4–5 weeks old, BALB/CNu) were injected subcutaneously with PANC‐1 cells (106 cells in 100 μL phosphate‐buffered saline). Tumour length and width were measured regularly, and after 30 days, mice were sacrificed under animal welfare guidelines.

### 2.12 RNAseq and bioinformatic analyses

For RNA sequencing, differential expression analysis of three groups (three biological replicates per condition) was performed using the DESeq2 R package (1.20.0). Benjamini and Hochberg's approach were used to adjust the resulting *p*‐values for controlling the false discovery rate. *p*_adj_≤0.05 and |log2(foldchange)| ≥1 were set as the threshold for significantly differential expression.^20^

### 2.13 Statistical analysis

Statistical analyses were performed using SPSS 26.0 (IBM) and GraphPad Prism 9.0 software. The number of samples (n) are described in detail for each figure. Data are presented as the mean ± SEM. The Student two‐tailed *t* test was used to analyse the differences between groups and presented them in the form of mean ± SD. The Kaplan–Meier method was used to test the OS curves of IHC data. *p* < 0.05 was defined as statistically significant.

## 3 RESULTS

### 3.1 *TFEB* is overexpressed in pancreatic cancer tissues and cells

**FIGURE 1:**
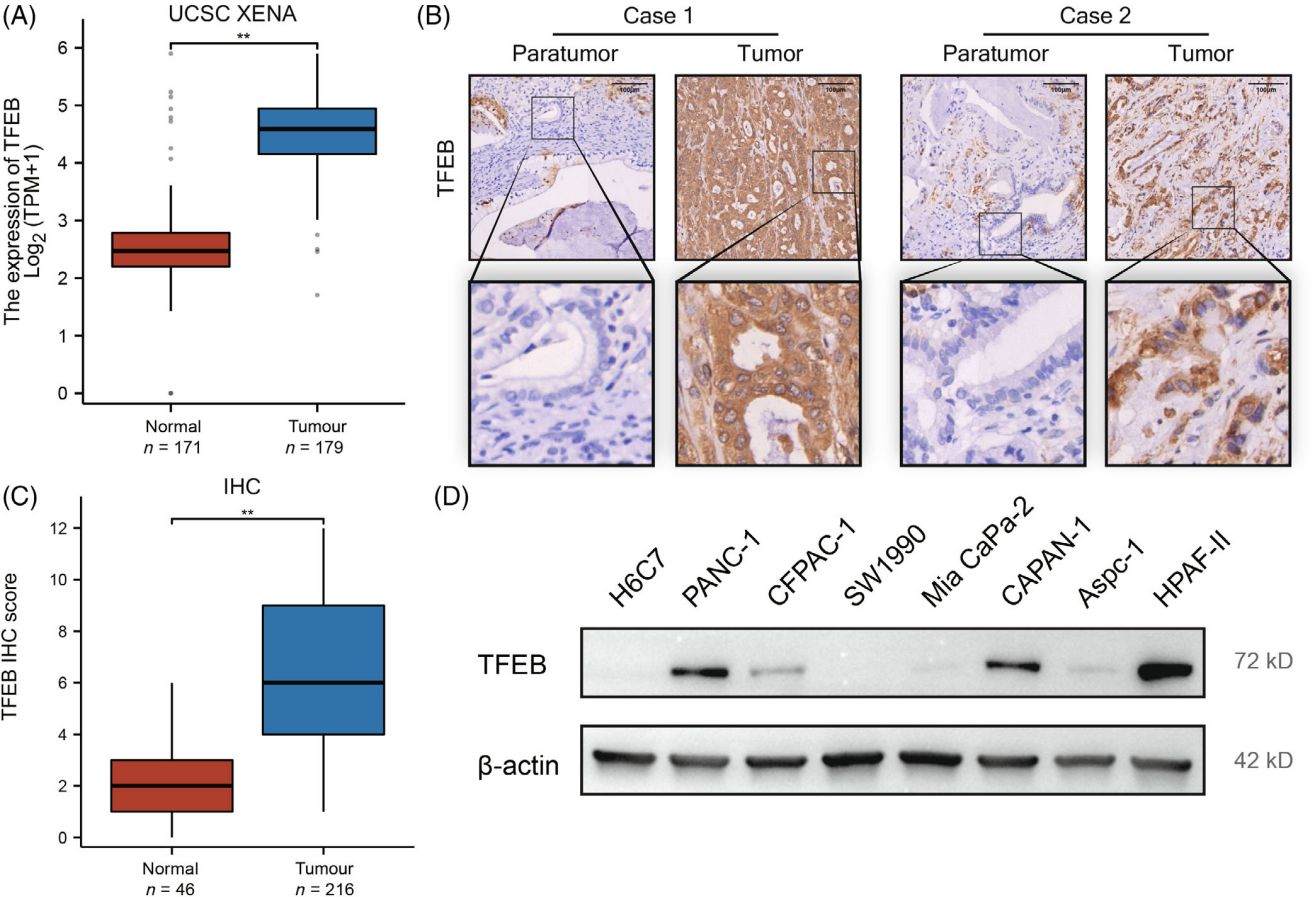
TFEB is overexpressed in pancreatic cancer tissues and cells. (A) TFEB expression data were derived from RNAseq data in TPM format for TCGA and GTEx uniformly processed by UCSC XENA via the Toil process,^38^ extracting data corresponding to TCGA in (pancreatic cancer) and corresponding normal tissue data in GTEx. (B) Typical picture of IHC staining of tumour tissue and its corresponding paraneoplastic tissue. (C) Comparative box line plots of IHC scores of 43 tumour tissues and 43 paraneoplastic tissues, and statistical analysis of the results. (D) WB analysis of TFEB content in individual cell lines of pancreatic cancer (*n* = 3 independent experiments) (*, *p* < 0.05; **, *p* < 0.01).

To explore the relation between TFEB and pancreatic cancer, we used the UCSC‐XENA database to determine the TFEB expression levels in pancreatic cancer and adjacent tissues. The results showed that the average expression of TFEB in 179 pancreatic cancer tissues was significantly higher than that in 171 adjacent tissues (Figure 1A). This was further verified by pairing the pancreatic cancer with the adjacent tissue in 10 patients at our centre. We found that TFEB was significantly upregulated in cancer compared with that in the adjacent tissue, and the nucleus was stained as well (Figure 1B). Furthermore, the staining and scoring of 43 adjacent tissues and 43 cancer tissue microarrays showed that the staining score in tumours was significantly higher than that in adjacent tissues (Figure 1C). To further explore the expression of TFEB in pancreatic cancer and normal pancreatic cells, the protein expression of normal pancreatic immortalised cells HPDE6‐C7 (H6C7) was verified by western blot analysis in various pancreatic cancer cell lines. The expression of TFEB in almost all pancreatic cancer cell lines was higher than that in normal pancreatic H6C7 cells (Figure 1D).

### 3.2 TFEB is essential for the proliferation and metastasis of PCCs

**FIGURE 2:**
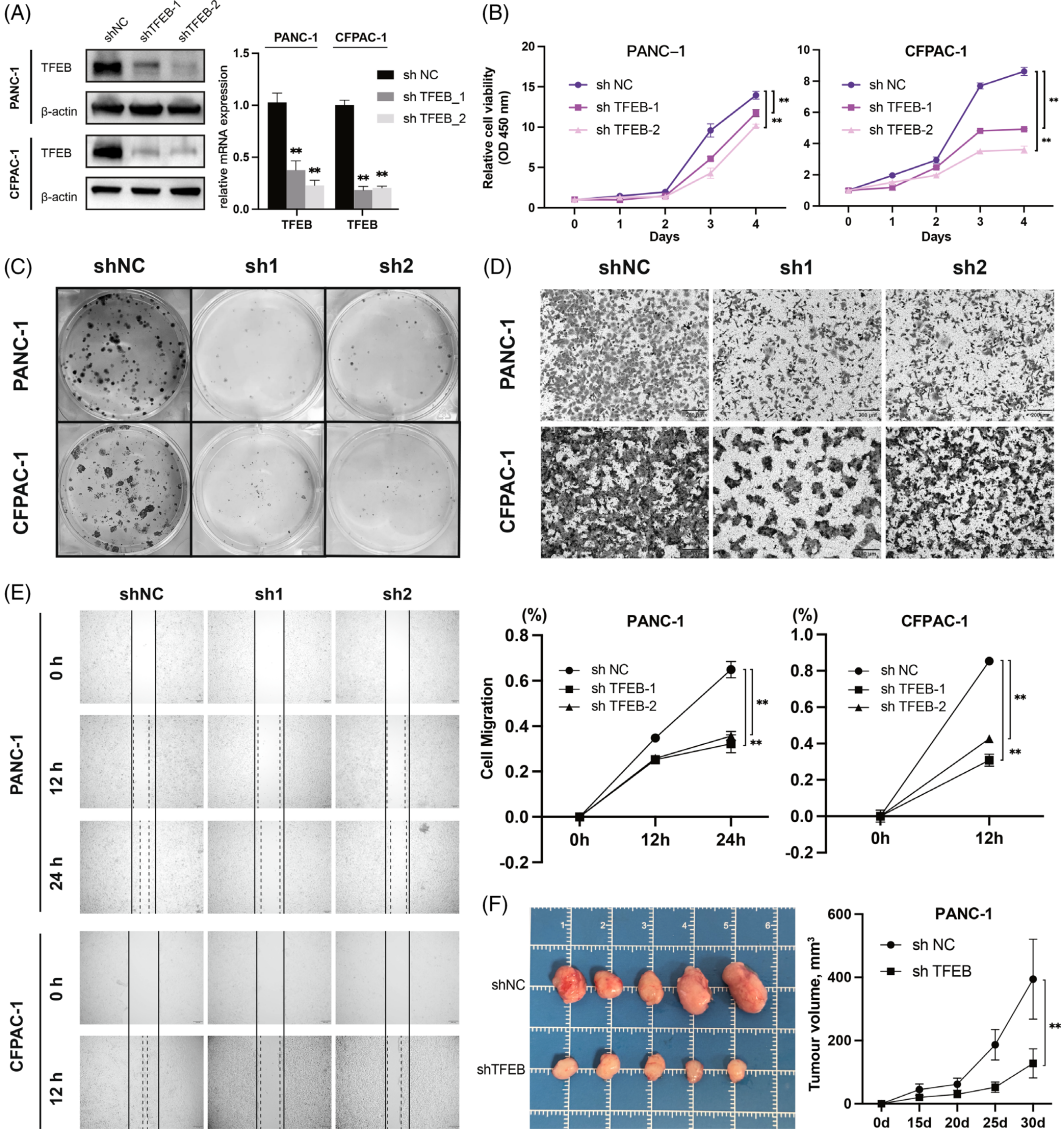
TFEB is essential for the proliferation and metastasis of PCCs. (A) Western blot and RT‐qPCR demonstrated the expression of TFEB in control cells versus TFEB knockdown stable transplants (*n* = 3 independent experiments). (B, C) Cell proliferation CCK8 assay (*n* = 5 biological replicates across three independent experiments) and cell clone formation assay (*n* = 3 independent experiments) demonstrating changes in cell proliferation after TFEB knockdown. (D) Cell migration transwell assay demonstrating altered cell migration ability after TFEB knockdown (*n* = 3 independent experiments). (E) Cell migration scratch assay demonstrating altered cell migration ability after TFEB knockdown (*n* = 3 independent experiments). (F) In vivo mouse tumorigenesis experiments, tumour volume change curves with mouse tumour pictures, top row shows the tumorigenesis of PANC‐1 cells in the shNC group, and the bottom row positions the tumorigenesis of PANC‐1 cells in the shTFEB group (*n* = 5 mice per group) (*, *p* < 0.05; **, *p* < 0.01). PCCs, pancreatic cancer cells.

To investigate the effect of high TFEB expression on the biological behaviour of pancreatic cancer cells, we transfected PANC‐1 and CFPAC‐1 cells with two TFEB shRNAs. The expression in the cells was verified by RT‐qPCR and western blot analysis (Figure 2A). The validation results showed that compared with the TFEB expression of the control group, the TFEB expression of the knockdown groups of PANC‐1 and CFPAC‐1 cells was significantly downregulated; therefore, they were used for follow‐up experiments. CCK8 and clone formation experiments showed that *TFEB* knockdown cells exhibited reduced proliferation and clone‐forming abilities (Figure 2B,C). Transwell and scratch assays revealed that *TFEB* knockdown significantly reduced cell migration (Figure 2D,E). Likewise, in PANC‐1 cells, cell proliferation and migration were significantly enhanced by overexpression of TFEB (Figure S1). To further validate the results of the in vitro assay, we established a xenograft model in BALB/c nude mice and analysed the in vivo effects of endogenous TFEB. The results showed that the ability of *TFEB* knockdown cells to form tumours in nude mice was significantly reduced, and their tumorigenesis rate and size were significantly lower than those in the control group (Figure 2F). Both in vivo and in vitro experiments showed that TFEB plays an important role in the proliferation and metastasis of pancreatic cancer.

### 3.3 Knockdown of *TFEB* imposes restrictions on catabolism and utilisation of BCAAs

**FIGURE 3:**
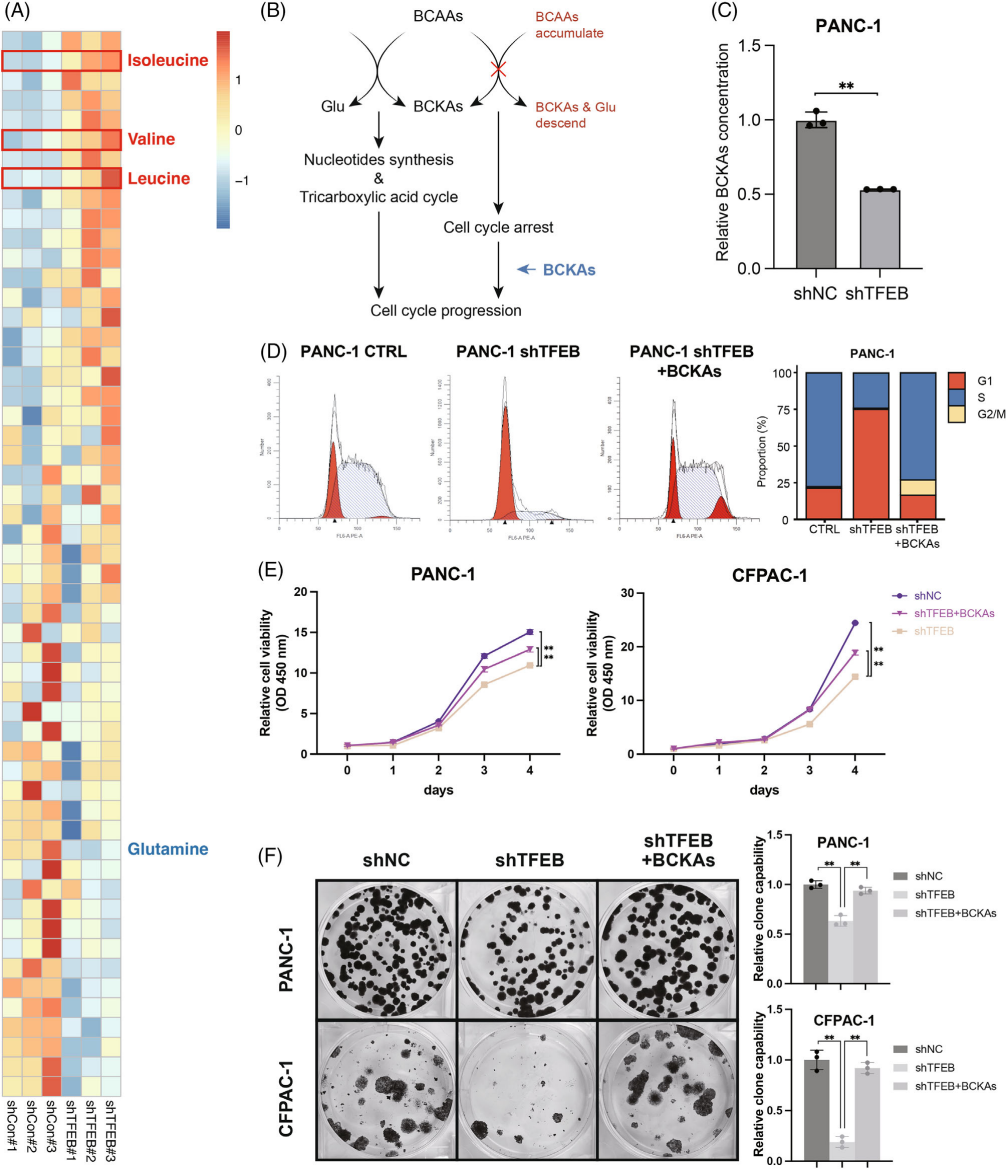
Knockdown of *TFEB* imposes restrictions on catabolism and utilisation of BCAAs. (A) Differential amino acid heatmap, after TFEB knockdown compared to the control group (*n* = 3 biological replicates). (B) Pattern diagram, the left half shows the way in which branched‐chain amino acids are catabolised and utilised, which in turn promotes cell cycle progression; the right half shows the way in which the cell cycle receives a block when branched‐chain amino acid utilisation is impaired. (C) Elisa detection of BCKAs in PANC‐1 cells after TFEB knockdown (*n* = 3 biological replicates across three independent experiments). (D) Detection of cell cycle changes in cells cultured in normal medium after TFEB knockdown compared to control PANC‐1 cells, and in cells cultured with BCKAs supplementation after TFEB knockdown (*n* = 3 independent experiments). (E, F) Cell proliferation CCK8 assay (*n* = 5 biological replicates across three independent experiments) and cell clone formation assay (*n* = 3 independent experiments) demonstrating changes in cell proliferation among cells cultured in normal medium after TFEB knockdown, and in cells cultured with BCKAs supplementation after TFEB knockdown compared to control PANC‐1 cells (*, *p* < 0.05; **, *p* < 0.01). BCAAs, branched‐chain amino acids.

Amino acids are essential nutrients in PCCs and are an indispensable nutrient supply for growth and proliferation. To explore the amino acid changes induced by the key regulatory gene of autophagy, *TFEB*, after its knockdown, we conducted a targeted metabolomic study and found that BCAAs, including leucine, isoleucine and valine, were significantly upregulated after *TFEB* knockdown (Figure 3A). However, it is interesting to note that glutamine, a catabolite product of glutamate and BCAAs, was abnormally downregulated, which may mean that the catabolism of BCAAs was retarded after *TFEB* knockdown and consequently, BCAAs accumulated (Figure 3B).

To verify whether the accumulation of BCAAs was caused by catabolic restriction, we used an ELISA kit to measure the concentration of branched‐chain keto acids (BCKAs), which are metabolites of BCAAs. BCKAs were downregulated after *TFEB* knockdown (Figure 3C). Since one of the important roles of BCKAs is to support cell division and proliferation as raw materials for cell synthesis, we performed cell cycle assays, which showed that after *TFEB* knockdown, the cell cycle was significantly arrested in the G1 phase (pre‐phase of DNA synthesis) and could not enter the S phase (DNA synthesis phase). Addition of BCKAs restored cell cycle progression (Figure 3D). CCK8 and clone formation assays showed that the addition of BCKAs during cell culture with *TFEB* knockdown reversed the inhibition of cell proliferation (Figure 3E,F). These results verified that *TFEB* knockdown led to impaired utilisation by blocking the catabolism of BCAAs. The addition of BCAAs' catabolic products can reverse the arrest effect caused by *TFEB* knockdown so that cancer cells can return to the normal cell cycle and resume rapid growth and proliferation.

### 3.4 TFEB dominates the utilisation of BCAAs by regulating BCAT1 and their clinical correlation

**FIGURE 4:**
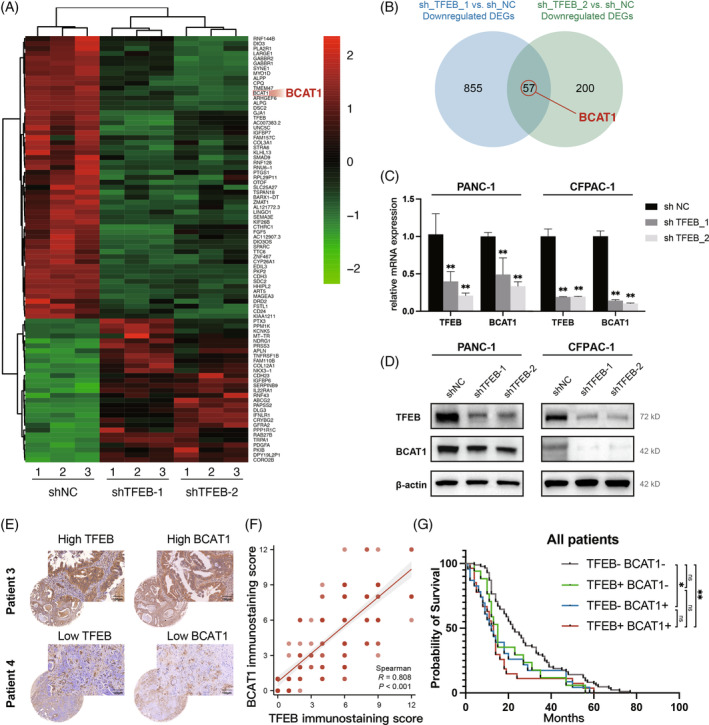
TFEB dominates the utilisation of BCAAs by regulating BCAT1 and their clinical correlation. (A) The 86 variant significant genes between the CTRL group and the two TFEB knockdown groups (*n* = 3 biological replicates per group) shown by the RNAseq results were clustered and displayed with the heat map. The “pheatmap” package (version 1.0.12) was used, with the method of “ward.D.” (B) The Wayne diagram shows the differential genes screened by RNAseq, with two differential genes in the knockdown and control groups selected and selected for intersection, in which BCAT1 was found. (C, D) RT‐qPCR and western blot to analyse the changes of BCAT1 after the knockdown of TFEB (*n* = 3 independent experiments). (E) Typical IHC plot of high/low expression of TFEB versus BCAT1 in patient specimens. (F) Staining extent correlation analysis between TFEB and BCAT1 (*n* = 218, *R* = 0.808, *p* < 0.001, Spearman's correlation analysis). (G) Kaplan–Meier analysis of survival of patients with different expression profiles of TFEB and BCAT1, including TFEB‐/BCAT1‐ (*n* = 66), TFEB+/BCAT1‐ (*n* = 59), TFEB‐/BCAT1+ (*n* = 38), TFEB+/BCAT1+ (*n* = 55) (*, *p* < 0.05; **, *p* < 0.01). BCAAs, branched‐chain amino acids.

To explore the molecular mechanism by which TFEB regulates BCAAs catabolism, we performed transcriptome sequencing of PANC‐1 cells to explore the differences in gene expression between the control and *TFEB* knockdown groups. *BCAT1* was downregulated at both *TFEB* knockdown sites (Figure 4A,B). BCAT1 catalyses the first step of BCAAs catabolism, producing glutamate and branched‐chain ketoacids.^21^ RT‐qPCR and western blot analyses confirmed that the RNA content and protein expression of BCAT1 were reduced in *TFEB*‐knockdown PANC‐1 and CFPAC‐1 cells (Figure 4C,D).

To further validate the relation between TFEB and BCAT1 expression in patient tissue samples, we stained tissue sections by immunohistochemistry and multiplied the staining depth and staining area to obtain the staining intensity area score (H‐score), and then intercepted the median value to classify the patients, as shown in the figure demonstrating a representative image of IHC staining (Figure 4E). Spearman's correlation analysis by staining intensity area assignment showed that TFEB expression was significantly correlated with BCAT1 expression (Figure 4F). In addition, we performed a combined prognostic analysis for four patients with high/low TFEB expression and high/low BCAT1 expression, and the Kaplan–Meier survival curves showed that patients with double‐positive TFEB and BCAT1 (high‐expression) were significantly correlated with worse overall survival, whereas those with double‐negative TFEB and BCAT1 (low‐expression) were associated with relatively better overall survival (Figure 4G). Besides, we explored the relationship between TFEB/BCAT1 expression and the clinicopathological features of pancreatic cancer. In our clinical data, we found that the high level of TFEB correlated with bigger tumour size (Tables S1 and S2), which also implies its promising clinical therapeutic application.

### 3.5 BCAT1 is regulated by TFEB at transcriptional level

**FIGURE 5:**
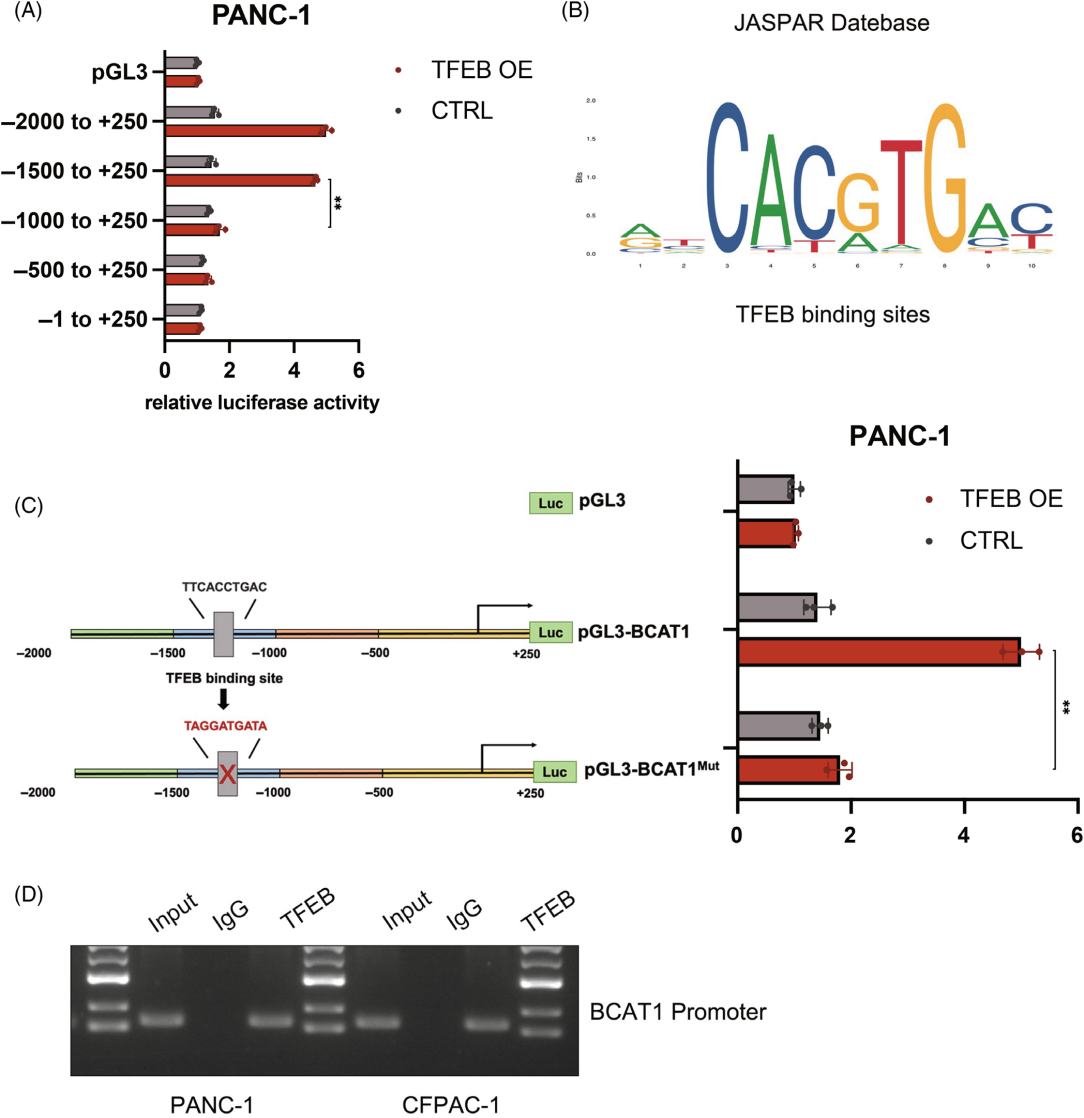
BCAT1 is regulated by TFEB at transcriptional level. (A) Schematic showing potential binding sites for TFEB on BCAT1. (B) The JASPAR database predicts binding site sequences for TFEB. (C) Effect of TFEB on the luciferase activity of the BCAT1‐dual luciferase reporter gene. (D) Typical CHIP assay image with TFEB antibody (*n* = 3 independent experiments) (*, *p* < 0.05; **, *p* < 0.01).

We explored the mechanism by which TFEB regulates BCAT1 expression. TFEB is a basic helix–loop–helix transcription factor whose many functions are achieved through transcriptional level regulation,^22^ so we speculate that TFEB may directly regulate *BCAT1* expression at the transcriptional level. Transcriptional assays were performed using a transiently transfected reporter construct containing five regions upstream of the *BCAT1* transcription start site. After TFEB co‐transfection, only the −1500 to −1000‐bp region significantly upregulated the luciferase signal (Figure 5A). In mammalian cells, the promoter regions of many genes share one or more 10‐base pair sequence (5′‐GTCACGTGAC‐3′), which was named the CLEAR element. The CLEAR consensus sequence contains an E‐box sequence targeted by TFEB.^23^ We used the JASPAR database^24^ to predict the binding sites between *TFEB* and the *BCAT1* promoter region and screened for putative binding sites in this region (Figure 5B,C). Subsequent luciferase assays suggested that mutation of the motif blocked TFEB‐induced transcriptional upregulation. Chromatin immunoprecipitation assays further confirmed that *TFEB* interacted with the −1500 to −1000‐bp region up‐stream of *BCAT1* in PANC‐1 and CFPAC‐1 cells (Figure 5D). These results indicate that *TFEB* binds to the *BCAT1* promoter and promotes the transcription of *BCAT1*.

### 3.6 Therapeutic insight of targeting TFEB and BCAAs

**FIGURE 6:**
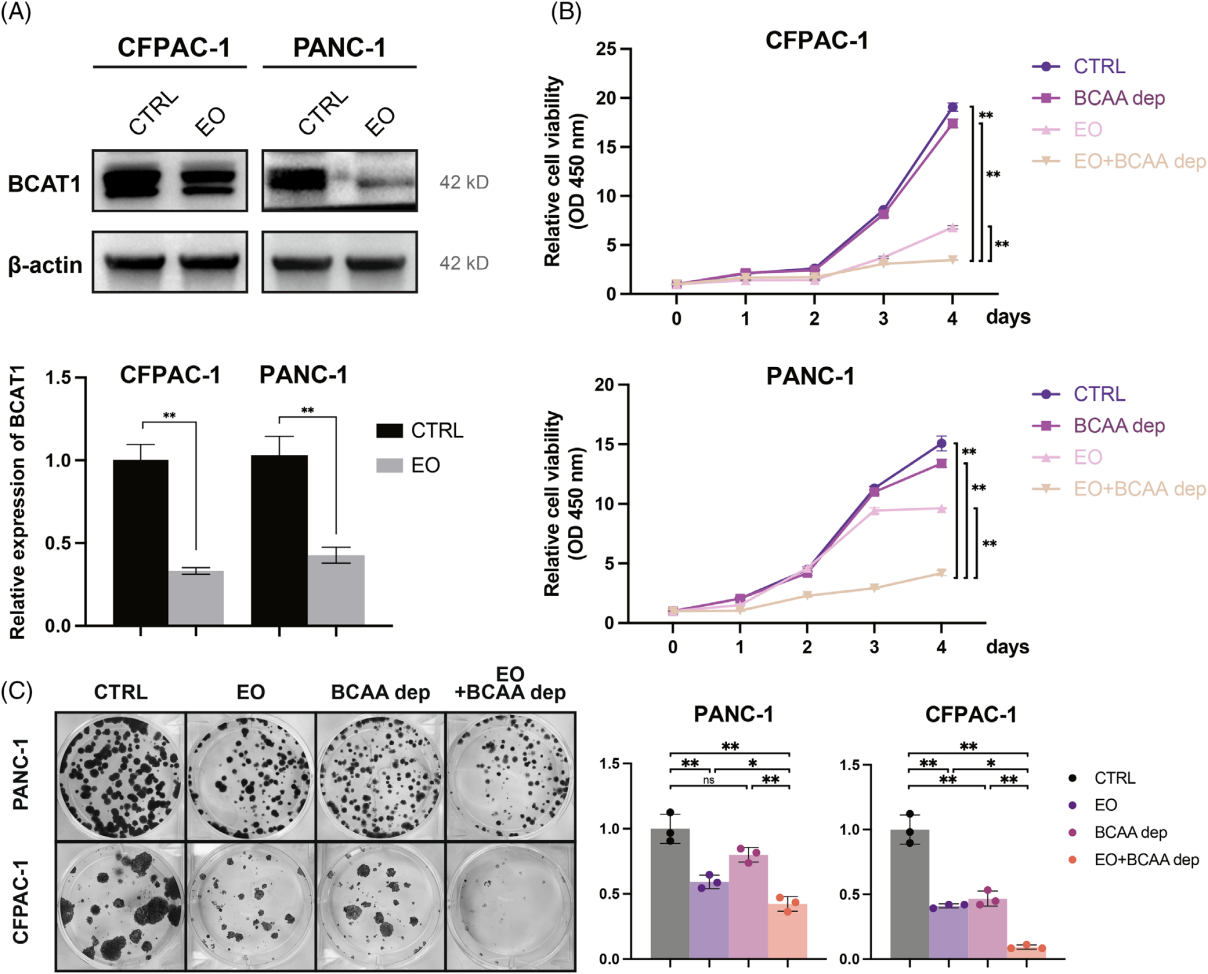
Therapeutic insight of targeting TFEB and BCAAs. (A) Western blot and RT‐qPCR demonstrated BCAT1 expression in PANC‐1 cells and CFPAC‐1 cells after the addition of Eltrombopag (EO) (*n* = 3 independent experiments). (B, C) CCK8 assay (*n* = 5 biological replicates across three independent experiments) and cell clone formation assay (*n* = 3 independent experiments) were performed to analyse cell proliferation after the addition of Eltrombopag (EO) and after culture with BCAA deprivation medium (*, *p* < 0.05; **, *p* < 0.01). BCAAs, branched‐chain amino acids.

Recently, EO was reported to inhibit TFEB function by binding to the basic helix–loop–helix‐leucine zipper structural domain of TFEB, preventing it from binding to DNA.^25^ After adding EO, the expression of BCAT1 was verified by western blot analysis and RT‐qPCR and was found to be reduced (Figure 6A). To investigate the effects of TFEB inhibition and BCAA deprivation on cell proliferation, we performed cell proliferation, CCK8 and clone formation assays, both of which showed significant inhibition of cell proliferation when TFEB function was inhibited by EO (Figure 6B,C). Although culturing with BCAA deprivation medium alone did not have a significant inhibitory effect on cell proliferation (Figure 6C), the combined use of EO under BCAA deprivation culture conditions significantly inhibited cell proliferation, with a stronger inhibitory effect than EO alone.

## 4 DISCUSSION

Pancreatic cancer is a highly malignant tumour, and in recent years, the increasing incidence of pancreatic cancer has led to a gradual increase in therapeutic research on pancreatic cancer. However, most studies focus on gene targets to treat pancreatic cancer by targeting pancreatic cancer‐specific genes to alter cell signalling. However, increasing evidence suggests that by targeting the unique metabolic signature of pancreatic cancer, new strategies can be developed to treat pancreatic cancer while minimising damage to healthy cells.^26^

Autophagy plays a bidirectional role in cancer development. In the early stages of cancer development, autophagy can prevent cancer from occurring by cleaning up harmful substances accumulated in the cell. However, autophagy protects cancer cells from surviving in various unfavourable conditions.^27^ TFEB can improve the ability of cancer cells to cope with various environmental stresses due to its ability to maintain cellular homeostasis through the regulation of autophagy. Cancer cells regulate the activity through an energy demand‐directed feedback mechanism. Thus, when there is an energy demand, *TFEB* is activated and promotes autophagy to provide an additional energy supply to cancer cells, which promotes cancer cell survival and cancer progression; thus, it is considered an oncogenic gene.^28^ It has been shown that *TFEB* is able to affect the cell cycle and thus promote epithelial cell proliferation by regulating CDK4/CyclinD1/Rb pathway.^29^ In melanoma, *TFEB* has also been reported to be able to affect cell metabolism and proliferation by regulating the DUSP‐1/ERK1/2 pathway.^30^ However, the regulatory mechanisms of *TFEB* in pancreatic cancer remain unclear.

TFEB was found to be highly expressed relative to paraneoplastics in various cancer cell lines. Our clinical samples also showed high expression of TFEB in pancreatic cancer, which is in line with the conclusions obtained from the analysis of data from TCGA and GTEx databases (Figure 1). Our study verified its role in promoting pancreatic cancer cell proliferation, metastasis and tumorigenesis in vivo and in vitro (Figure 2). TFEB has been reported to be able to control cellular metabolic patterns and promotes tumour progression by inducing autophagy.^31^ However, the unique physiological characteristics of pancreatic cancer, including higher interstitial pressure and thicker connective tissue, have led to a shift in metabolic mode that is different from that of other tumours, and the use of amino acids as an energy supply, rather than just as a constituent raw material for proteins, is one of the characteristics of pancreatic cancer.^9^ Autophagy has been reported to be able to maintain the supply of amino acids required by cells. However, whether amino acid utilisation and functional shifts in pancreatic cancer have a relevant regulatory relation with autophagy and TFEB remains to be explored.

Since TFEB can regulate autophagy, which is closely related to intracellular amino acid levels, the decrease in autophagic flux after *TFEB* knockdown decreases the availability of amino acids produced by protein autophagic degradation.^32^ Interestingly, using a metabolomic assay, we found that intracellular BCAA levels were upregulated after *TFEB* knockdown. Moreover, we found that additional supplementation of BCAAs was unable to reverse the proliferative hindrance brought by the knockdown of TFEB (Figure S2). This suggests that knockdown of TFEB was able to impede the utilisation of BCAAs in PCCs, which resulted in reduced proliferation. The upregulation of BCAA levels did not result in a tumour‐promoting effect, consistent with its tumour growth‐promoting function, and cells after *TFEB* knockdown inhibited growth and proliferation (Figure 3). In addition, glutamine, which is produced from the catabolite products of BCAAs, was abnormally downregulated. This suggests that the downregulation of *TFEB* may have simultaneously caused the inhibition of the BCAA degradation and utilisation pathways, which resulted in a paradoxical elevation and impaired utilisation of BCAAs. To verify this, we assayed the levels of BCKAs in PANC‐1 cells after *TFEB* knockdown and found a significant downregulation. The metabolites of BCAAs, including BCKAs and glutamate, are the raw materials for nucleotide synthesis and the tricarboxylic acid cycle, which provide the raw materials for cellular synthesis and energy supply,^33^, ^34^ and once deficient, the cell cycle is stalled in the pre‐synthesis phase (G1 phase), owing to the lack of raw materials, which blocks cell division and proliferation. Meanwhile, other pro‐proliferating signalling pathways, including MAPK, PI3K pathways, which are closely related to cell proliferation, did not show significant changes after TFEB knockdown (Figure S3). We observed that knockdown of TFEB caused cell cycle arrest, most likely associated with an inadequate supply of raw materials for nucleotide synthesis. This was reversed by the addition of the BCAAs metabolite BCKAs, which also proved that the BCAA metabolic pathway is key to TFEB‐mediated cell cycle changes and cell proliferation in pancreatic cancer rather than other pro‐cancer signalling pathways (Figure 3E,F).

To address the molecular mechanism of TFEB regulation of BCAAs catabolism, we collected PANC‐1 cells from *TFEB* knockdown and control groups and analysed the differences using RNAseq. Among these, changes in BCAT1 expression attracted our attention. BCAT1 and BCAT2 are two isoenzymes, both BCAA transaminases, which catalyse the initial transamination step of BCAA catabolism and produce BCKAs and glutamate.^35^ According to the transcriptome sequencing results, the expression of BCAT1 was downregulated upon *TFEB* knockdown (Figure 4). Subsequently, its function in catalysing the catabolism of BCAAs was inhibited, which explains the accumulation of BCAAs and the decline in BCKAs. However, the mechanism by which TFEB regulates BCAT1 is not clear; therefore, we explored this further. Since TFEB is a transcription factor, we hypothesised that it drives BCAT1 synthesis by directly binding to the BCAT1 promoter region at the transcriptional level. To verify this, we used the JASPAR database to predict the potential binding sites between *TFEB* and the *BCAT1* promoter region and identified multiple possible binding sites. Chromatin immunoprecipitation experiments using a TFEB antibody showed that TFEB was able to bind to the *BCAT1* promoter region. The dual‐luciferase assay also showed that as the amount of TFEB increased, the amount of BCAT1 increased accordingly. This further validated the transcriptional regulation of BCAT1 by TFEB, which also implied that pancreatic cancer cells acquire metabolic shifts favouring their survival through the TFEB‐BCAT1‐BCAAs pathway (Figure 5).

IHC staining and combined prognostic analysis of clinical samples from patients with pancreatic cancer revealed that patients with double positivity (high expression) for TFEB and BCAT1 tended to have a poorer prognosis. This may be related to the fact that its TFEB‐BCAT1‐BCAAs functional pathway is more active, leading to the occurrence of metabolic changes that favour cancer cell survival and progression. Targeting this pathway may provide new countermeasures for pancreatic cancer treatment.

It has been reported that cancer development can be delayed by restricting dietary BCAA content. However, BCAA‐associated cancer treatments remain controversial because of the different prognoses of HCC mouse models supplemented with BCAAs and patients with hepatocellular carcinoma.^36^ To investigate the effect of BCAA deprivation on pancreatic cancer, we cultured cells after excluding exogenously supplied BCAAs in the BCAAs deprivation medium and found that the BCAAs deprivation group demonstrated some proliferation suppression relative to the control group, but the difference was not statistically significant (Figure 6). Studies have shown that pancreatic cancer cells themselves can take up extracellular proteins through macropinocytosis, which are degraded and used to fulfil glutamine requirements while leaving behind BCAAs that cause accumulation.^9^ It also retains a certain amount of amino acid reserves through the lysosomal storage of amino acids,^37^ which may be the reason why blocking the supply of exogenous BCAAs is still not able to cut off the supply and utilisation of BCAAs in pancreatic cancer cells. For this reason, together with the potentially deleterious reactions to long‐term deprivation of essential amino acids, therapies such as a BCAA‐deprived diet alone are not currently seen as a beneficial therapeutic direction for pancreatic cancer. It is important to block the endogenous BCAAs utilisation pathway to prevent the progression of pancreatic cancer brought about by BCAA utilisation in pancreatic cancer.

**FIGURE 7:**
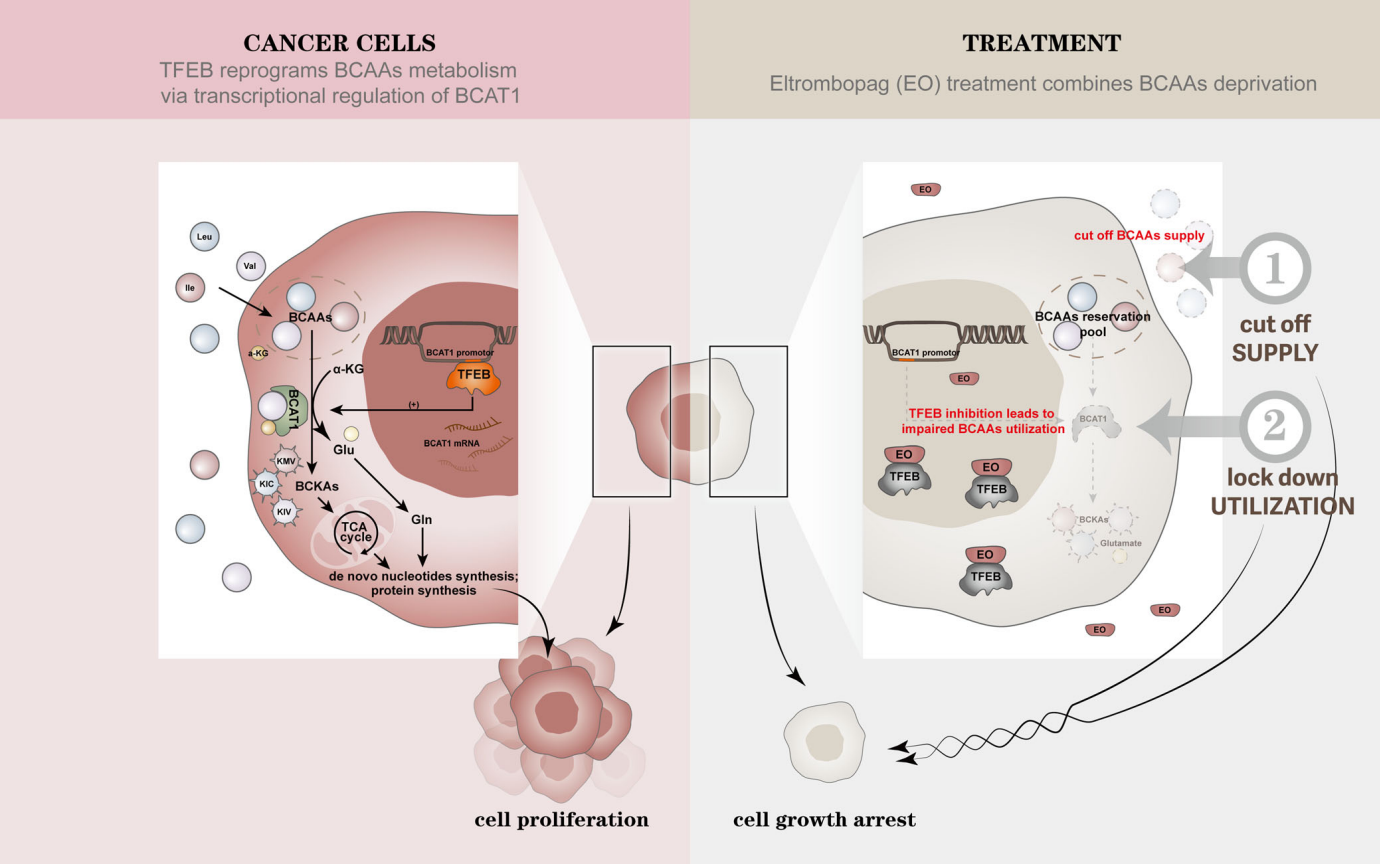
TFEB reprograms branched‐chain amino acid metabolism via transcriptional regulation of *BCAT1* and the target strategy. In this schematic diagram, the left side shows the mechanism by which TFEB reprograms the metabolism of BCAAs in tumour cells through transcriptional regulation of *BCAT1*, and the right side shows the combination of exogenous cut‐off of BCAAs supply and endogenous cut‐off of BCAAs utilisation to exert maximal inhibition of tumour cells. In pancreatic cancer cells, TFEB can regulate the metabolism and utilisation of BCAAs through transcriptional regulation of *BCAT1*, which converts BCAAs and α‐keto acids into glutamate and BCKAs, which are further converted into glutamine for nucleotide synthesis and protein synthesis; whereas BCKAs enter the tricarboxylic acid cycle to provide raw materials for nucleotide and protein production, all of which are indispensable raw materials for tumour cell proliferation. Regarding therapeutic strategies, BCAAs deprivation (culture medium) can cut off the external supply of BCAAs. However, since cancer cells can retain a certain amount of branched‐chain amino acid reserves through phagocytosis of large proteins and uptake of extracellular protein hydrolysates, it is necessary to further target their metabolic pathways to cut off the supply and utilisation of branched‐chain amino acids by pancreatic cancer cells. Targeting TFEB can block the transcription of *BCAT1*, thereby blocking the metabolic utilisation of BCAAs. Blocking its supply exogenously by BCAAs deprivation, in conjunction with the TFEB inhibitor eltrombopag (EO) to block endogenous utilisation, maximises the inhibition of pancreatic cancer progression by blocking branched‐chain amino acid utilisation in pancreatic cancer. BCAAs, branched‐chain amino acids.

Recently, it was reported that the small‐molecule drug EO can inhibit the transcriptional regulatory function of TFEB by binding to the DNA‐binding site on TFEB and hindering its combination with DNA.^25^ To investigate whether EO treatment of pancreatic cancer cells could reduce the tumour‐promoting effects of endogenous BCAAs catabolism and utilisation, we compared the proliferation of pancreatic cancer cells with and without EO. Both the cell proliferation CCK8 assay and clone formation assay showed that the growth and proliferation of pancreatic cancer cells were inhibited by the addition of EO. When we used BCAAs deprivation medium in combination with EO treatment, the proliferation of pancreatic cancer cells was significantly inhibited by deprivation of exogenous BCAAs supply in conjunction with blocking the endogenous BCAAs utilisation pathway, showing maximised cancer inhibition compared with that in the exogenous deprivation or endogenous blockade alone (Figure 7).

Overall, this study found that TFEB directly regulates *BCAT1* expression at the transcriptional level, which in turn regulates the catabolism and utilisation of BCAAs. The combined use of exogenous BCAAs deprivation and endogenous BCAAs utilisation blockade can maximise the inhibitory effects on cancer. For example, the combined use of EO, a TFEB inhibitor based on BCAA dietary restriction, provides a new direction for targeting novel therapeutic modalities, such as metabolic reprogramming, in pancreatic cancer.

## AUTHOR CONTRIBUTIONS

Zheng Li, Xianjun Yu, Yi Qin and Xiaowu Xu conceived of the presented idea. Guixiong Fan, Desheng Jing and Junfeng Xu designed the experiments. Ting Wang, Qiangsheng Hu and Borui Li performed the experiments, analysed all data and draft the manuscript. Yuheng Hu, Qing Dang, Shunrong Ji, Chenjie Zhou and Qifeng Zhuo provided suggestions in experiments and manuscript drafting. All authors reviewed the manuscript, and made a final approval.

## FUNDING INFORMATION

This study was supported by National Natural Science Foundation of China (81972250, 82173281, 82141129, 82172948), Excellence project of Shanghai Municipal Health Commission (20224Z0006), Sailing Project of Science and Technology Commission of Shanghai Municipality (22YF1409000), Clinical Research Project of Health Industry of Shanghai Municipal Health Commission (20234Y0119).

## CONFLICT OF INTEREST STATEMENT

There are no conflicts of interest or financial ties to disclose.

## Supporting information


**FIGURE S1.** Overexpression of *TFEB* promotes the proliferation and metastasis of PCCs. (A) RT‐qPCR and western blot to demonstrate the expression of TFEB in control cells versus TFEB overexpression stable transplants (*n* = 3 independent experiments). (B, C) CCK8 assay (*n* = 5 biological replicates across three independent experiments) and cell clone formation assay (*n* = 3 independent experiments) demonstrating changes in cell proliferation after TFEB overexpression. (D) Cell migration transwell assay demonstrating altered cell migration ability after TFEB overexpression (*n* = 3 independent experiments). (E) Cell migration scratch assay demonstrating altered cell migration ability after TFEB overexpression (*n* = 3 independent experiments) (*, *p* < 0.05; **, *p* < 0.01).
**FIGURE S2.** Addition of BCAAs was incapable of reversing the inhibition of proliferation caused by *TFEB* knockdown. (A) Cell proliferation CCK8 assay demonstrating changes in cell proliferation after TFEB knockdown (*n* = 5 biological replicates across three independent experiments). (B) Cell proliferation clone formation assay demonstrating changes in cell proliferation after TFEB knockdown (*n* = 3 independent experiments) (*, *p* < 0.05; **, *p* < 0.01).
**FIGURE S3.** *TFEB* knockdown showed no significant effect on *MAPK, PI3K* downstream signalling. (A) Change of *MAPK* downstream signalling in PANC‐1 cell lysates was determined using Western blot analysis through immunoblotting of Phospho‐ERK, ERK (*n* = 3 independent experiments). (B) Change of *PI3K* downstream signalling in PANC‐1 cell lysates was determined using Western blot analysis through immunoblotting of Phospho‐AKT, AKT (*n* = 3 independent experiments) (*, *p* < 0.05; **, *p* < 0.01).

**TABLE S1.** Clinicopathological features and correlation of TFEB expression in PDAC. TFEB^Low^, negative/weak TFEB expression; TFEB^High^, moderate/strong TFEB expression.

**TABLE S2.** Clinicopathological features and correlation of BCAT1 expression in PDAC. BCAT1^Low^, negative/weak BCAT1 expression; BCAT1^High^, moderate/strong BCAT1 expression.

## Data Availability

The data that support the findings of this study are available on request from the corresponding author. The data are not publicly available due to privacy or ethical restrictions.
