# Supplementary figures and images for: Transcription factor EB reprograms branched‐chain amino acid metabolism and promotes pancreatic cancer progression via transcriptional regulation of BCAT1
Source: Cell Prolif. 2024 Jun 27;57(11):e13694. doi: 10.1111/cpr.13694 (PMC11533072; doi:10.1111/cpr.13694)

**A**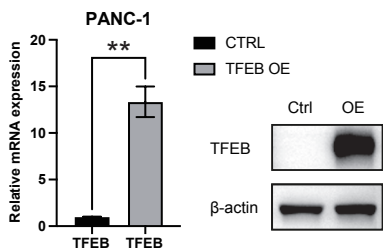**B**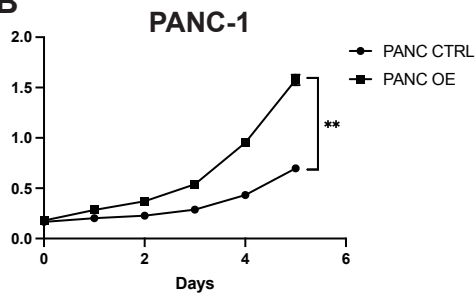**C**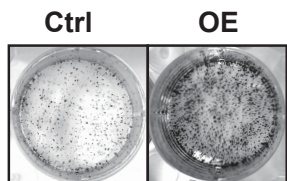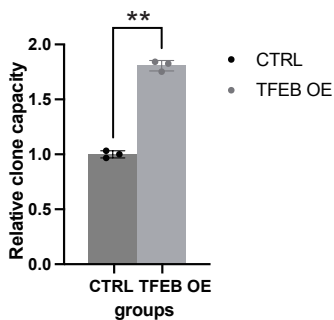**D**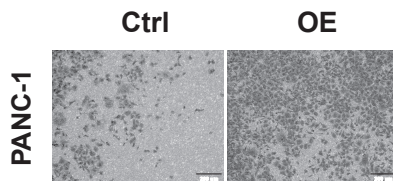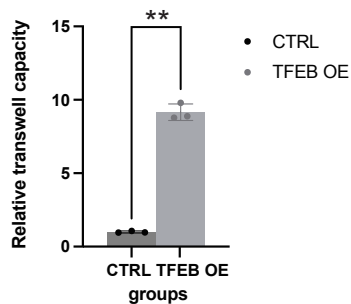**E**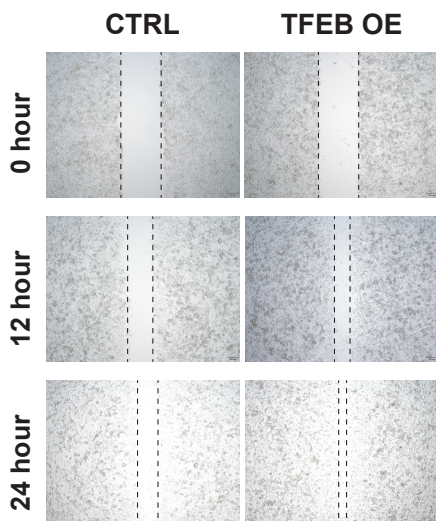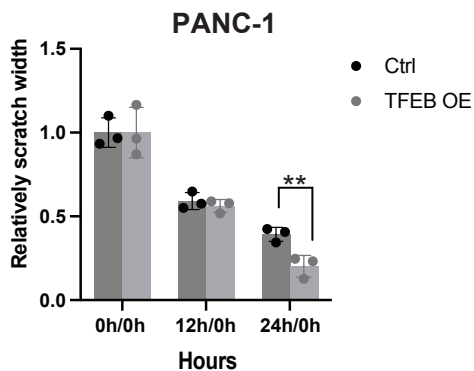

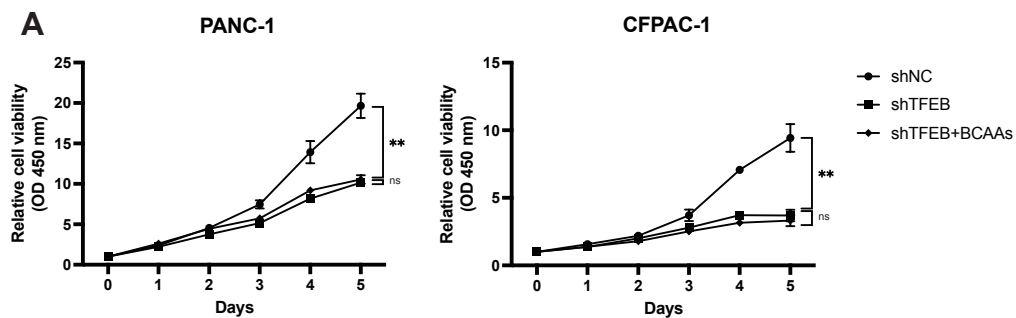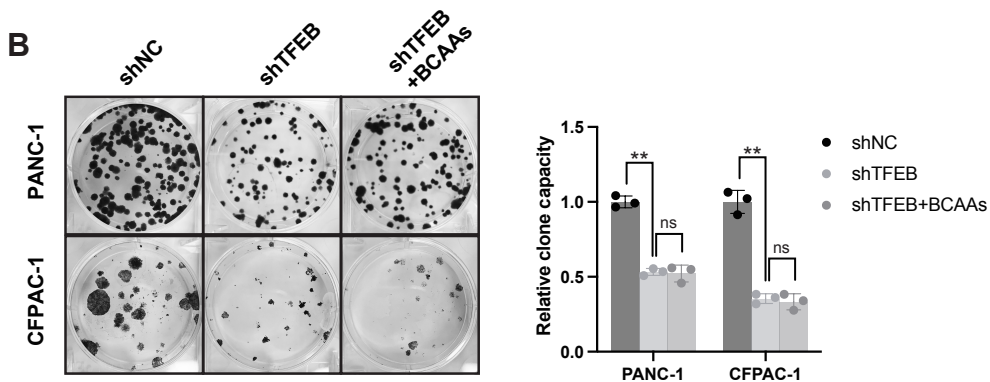

**A**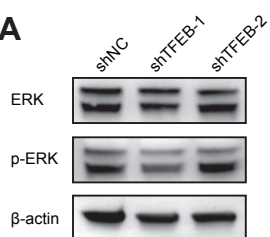**B**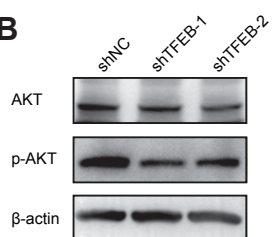

Supplement: Supplementary file 1 — FIGURE S1. Overexpression of TFEB promotes the proliferation and metastasis of PCCs. (A) RT‐qPCR and western blot to demonstrate the expression of TFEB in control cells versus TFEB overexpression stable transplants (n = 3 independent experiments). (B, C) CCK8 assay (n = 5 biological replicates across three independent experiments) and cell clone formation assay (n = 3 independent experiments) demonstrating changes in cell proliferation after TFEB overexpression. (D) Cell migration transwell assay demonstrating altered cell migration ability after TFEB overexpression (n = 3 independent experiments). (E) Cell migration scratch assay demonstrating altered cell migration ability after TFEB overexpression (n = 3 independent experiments) (*, p < 0.05; **, p < 0.01). FIGURE S2. Addition of BCAAs was incapable of reversing the inhibition of proliferation caused by TFEB knockdown. (A) Cell proliferation CCK8 assay demonstrating changes in cell proliferation after TFEB knockdown (n = 5 biological replicates across three independent experiments). (B) Cell proliferation clone formation assay demonstrating changes in cell proliferation after TFEB knockdown (n = 3 independent experiments) (*, p < 0.05; **, p < 0.01). FIGURE S3. TFEB knockdown showed no significant effect on MAPK, PI3K downstream signalling. (A) Change of MAPK downstream signalling in PANC‐1 cell lysates was determined using Western blot analysis through immunoblotting of Phospho‐ERK, ERK (n = 3 independent experiments). (B) Change of PI3K downstream signalling in PANC‐1 cell lysates was determined using Western blot analysis through immunoblotting of Phospho‐AKT, AKT (n = 3 independent experiments) (*, p < 0.05; **, p < 0.01). [file CPR-57-e13694-s002.pdf]
