# Supplementary material for: Transcription factor EB reprograms branched‐chain amino acid metabolism and promotes pancreatic cancer progression via transcriptional regulation of BCAT1
Source: Cell Prolif. 2024 Jun 27;57(11):e13694. doi: 10.1111/cpr.13694 (PMC11533072; doi:10.1111/cpr.13694)
Supplement: Supplementary file 2 — TABLE S1. Clinicopathological features and correlation of TFEB expression in PDAC. TFEBLow, negative/weak TFEB expression; TFEBHigh, moderate/strong TFEB expression. [file CPR-57-e13694-s003.docx]

| **Table S1. Clinicopathological features and correlation of TFEB expression in PDAC** | | | | | | | | |
| --- | --- | --- | --- | --- | --- | --- | --- | --- |
|  |  | | **TFEB-Low** | | **TFEB-High** | |  | |
| **Characteristics** | **No.** | | **score(-/+)(n=104)** | | **score(++/+++)(n=114)** | | **P Value** | |
| **Age(y)** |  | |  | |  | | 0.952 | |
| <60 | 78 | | 37 | | 41 | |  | |
| ≥60 | 140 | | 67 | | 73 | |  | |
| **Gender** |  | |  | |  | | 0.752 | |
| Female | 94 | | 46 | | 48 | |  | |
| Male | 124 | | 58 | | 66 | |  | |
| **Tumor size(cm)** |  | |  | |  | | **0.017** | |
| <4.0 | 144 | | 77 | | 67 | |  | |
| ≥4.0 | 74 | | 27 | | 47 | |  | |
| **Tumor differentiation** |  | |  | |  | | 0.416 | |
| Well | 19 | | 7 | | 12 | |  | |
| Moderate | 181 | | 90 | | 91 | |  | |
| Poor | 18 | | 7 | | 11 | |  | |
| **Lymph node status(stage)** | |  | |  | |  | | 0.881 |
| Negative(ⅡA) | 129 | | 61 | | 68 | |  | |
| Positive(ⅡB) | 89 | | 43 | | 46 | |  | |
| **Vessel Infiltration** |  | |  | |  | | 0.687 | |
| Negative | 85 | | 42 | | 43 | |  | |
| Positive | 133 | | 62 | | 71 | |  | |

TFEB^Low^--negative/weak TFEB expression; TFEB^High^--moderate/strong TFEB expression
