# Supplementary material for: Transcription factor EB reprograms branched‐chain amino acid metabolism and promotes pancreatic cancer progression via transcriptional regulation of BCAT1
Source: Cell Prolif. 2024 Jun 27;57(11):e13694. doi: 10.1111/cpr.13694 (PMC11533072; doi:10.1111/cpr.13694)
Supplement: Supplementary file 3 — TABLE S2. Clinicopathological features and correlation of BCAT1 expression in PDAC. BCAT1Low, negative/weak BCAT1 expression; BCAT1High, moderate/strong BCAT1 expression. [file CPR-57-e13694-s001.docx]

| **Table S2. Clinicopathological features and correlation of BCAT1 expression in PDAC** | | | | | | | | |
| --- | --- | --- | --- | --- | --- | --- | --- | --- |
|  |  | | **BCAT1-Low** | | **BCAT1-High** | |  | |
| **Characteristics** | **No.** | | **score(-/+)(n=104)** | | **score(++/+++)(n=114)** | | **P Value** | |
| **Age(y)** |  | |  | |  | | 0.287 | |
| <60 | 78 | | 41 | | 37 | |  | |
| ≥60 | 140 | | 84 | | 56 | |  | |
| **Gender** |  | |  | |  | | 0.761 | |
| Female | 94 | | 55 | | 39 | |  | |
| Male | 124 | | 70 | | 54 | |  | |
| **Tumor size(cm)** |  | |  | |  | | 0.063 | |
| <4.0 | 144 | | 89 | | 55 | |  | |
| ≥4.0 | 74 | | 36 | | 38 | |  | |
| **Tumor differentiation** |  | |  | |  | | 0.803 | |
| Well | 19 | | 12 | | 7 | |  | |
| Moderate | 181 | | 102 | | 79 | |  | |
| Poor | 18 | | 11 | | 7 | |  | |
| **Lymph node status(stage)** | |  | |  | |  | | 0.398 |
| Negative(ⅡA) | 129 | | 77 | | 52 | |  | |
| Positive(ⅡB) | 89 | | 48 | | 41 | |  | |
| **Vessel Infiltration** |  | |  | |  | | 0.941 | |
| Negative | 85 | | 49 | | 36 | |  | |
| Positive | 133 | | 76 | | 57 | |  | |

BCAT1^Low^--negative/weak BCAT1 expression; BCAT1^High^--moderate/strong BCAT1 expression
